# Cytoplasmic inheritance: The transmission of plastid and mitochondrial genomes across cells and generations

**DOI:** 10.1093/plphys/kiaf168

**Published:** 2025-04-30

**Authors:** Kin Pan Chung

**Affiliations:** Laboratory of Plant Physiology, Wageningen University & Research, Wageningen 6708 PB, the Netherlands

## Abstract

In photosynthetic organisms, genetic material is stored in the nucleus and the two cytoplasmic organelles: plastids and mitochondria. While both the nuclear and cytoplasmic genomes are essential for survival, the inheritance of these genomes is subject to distinct laws. Cytoplasmic inheritance differs fundamentally from nuclear inheritance through two unique processes: vegetative segregation and uniparental inheritance. To illustrate the significance of these processes in shaping cytoplasmic inheritance, I will trace the journey of plastid and mitochondrial genomes, following their transmission from parents to progeny. The cellular and molecular mechanisms regulating their transmission along the path are explored. By providing a framework that encompasses the inheritance of both plastid and mitochondrial genomes across cells and generations, I aim to present a comprehensive overview of cytoplasmic inheritance and highlight the intricate interplay of cellular processes that determine inheritance patterns. I will conclude this review by summarizing recent breakthroughs in the field that have significantly advanced our understanding of cytoplasmic inheritance. This knowledge has paved the way for achieving the first instance of controlled cytoplasmic inheritance in plants, unlocking the potential to harness cytoplasmic genetics for crop improvement.

## Introduction

From freedom to captivity,

From securing a home to losing their identity,

They were destined to live in servitude.

Yet they fought, fought hard for their genomic treasure,

Came out winners and then stayed on with pleasure.

Starting a review article with a poem may seem unconventional, yet this ode to plastids and mitochondria, excerpted from the book chapter Organellar Genomes of Flowering Plants, beautifully encapsulates the struggles and challenges faced by these endosymbionts (Choubey and Rajam 2015). The poem describes the transformation of once free-living cyanobacteria and alphaproteobacteria into plastids and mitochondria. Despite millions of years as endosymbionts, plastids and mitochondria still possess their own genomes, echoing remnants of their past autonomous existence. These cytoplasmic genomes encode proteins essential for cellular processes such as photosynthesis and respiration (Timmis et al. 2004; Greiner and Bock 2013). Given their critical role in survival, the proper inheritance of these organelles is paramount to ensure progeny inherits complete sets of cytoplasmic genomes during cell division and reproduction. While it might seem intuitive that mechanisms akin to nuclear genome inheritance would suffice for cytoplasmic genomes, this is far from reality. Cytoplasmic inheritance operates in a fundamentally different manner from nuclear inheritance, with most of Mendel's laws of inheritance not applying to cytoplasmic genomes (Birky 2001; Camus et al. 2022). The intriguing phenomenon of non-Mendelian inheritance of cytoplasmic genomes has captivated biologists for decades. What makes the “genomic treasure” of plastids and mitochondria so unique? Are there any laws governing the inheritance of cytoplasmic genomes?

## Laws of cytoplasmic inheritance

In 1976, Birky outlined general principles governing the inheritance of cytoplasmic genomes (Birky 1976). Among these, two unique processes (1) vegetative segregation and (2) uniparental inheritance, play major roles in shaping cytoplasmic inheritance in diverse species across kingdoms (Birky 1978). Notably, these 2 processes address the following key questions concerning cytoplasmic inheritance:

1. When a cell divides, how are the cytoplasmic genomes partitioned between daughter cells?
2. When organisms reproduce, how are the cytoplasmic genomes transmitted to offspring?

In this review, I will first introduce the concept of vegetative segregation and uniparental inheritance, followed by an in-depth examination of the molecular mechanisms underlying these processes.

## Vegetative segregation

Vegetative segregation describes the random partitioning of plastids and mitochondria during cell division, leading to the stochastic segregation of cytoplasmic genomes among daughter cells (Birky 1983; Chesser 1998).

Two key features of cytoplasmic genomes drive vegetative segregation are as follows:

### Relaxed replication of cytoplasmic genomes

Plastids and mitochondria contain multiple genome copies, with cells often housing numerous organelles, resulting in a variable range of genome copies per cell. The total number of genome copies per cell varies depending on species, cell type, and developmental stage. For instance, in Arabidopsis thaliana, plastid genome copy numbers in leaf cells are estimated to range from 1,000 to 1,700, while mitochondrial genome copies range from 300 to 450 in root tip cells and from 40 to 75 in flowers and stems (Shaver et al. 2006; Zoschke et al. 2007; Preuten et al. 2010; Schatz-Daas et al. 2022). Unlike the strictly regulated replication of nuclear genomes, cytoplasmic genomes replicate in a relaxed manner, allowing certain copies to be replicated more frequently, either by chance or through selective processes (Birky 1994; Birky 2001).

### Random partitioning of organelles during cell division

Plastids and mitochondria are dispersed throughout the cytoplasm, and in most cases, their partitioning into daughter cells occurs randomly (with exceptional cases discussed in later sections). There is no strict mechanism to guarantee an equal distribution of cytoplasmic genomes to each daughter cell (Birky 2001).

**Figure 1.**
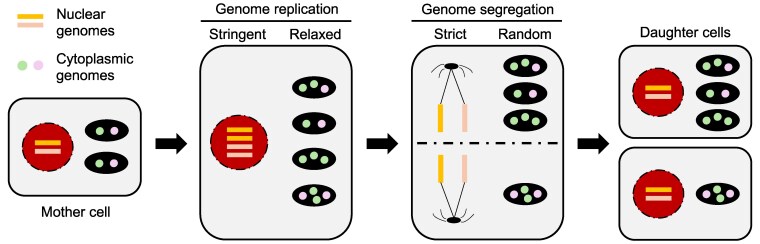
Nuclear genomes undergo strict genome replication and segregation during mitosis, ensuring that each daughter cell inherits an identical set of chromosomes. In contrast, cytoplasmic genomes undergo relaxed genome replication, altering their overall composition within the mother cell. This, in turn, affects the abundance and types of genomes that are passed on to daughter cells. During cell division, cytoplasmic organelles are randomly partitioned, resulting in daughter cells with distinct cytoplasmic genome compositions.

In sum, the stochastic segregation of cytoplasmic genomes between daughter cells sharply contrasts with the precise inheritance of the nuclear genome (Fig. 1) (McIntosh and Koonce 1989; Birky 1994).

## Uniparental inheritance

Uniparental inheritance describes the selective transmission of plastids and mitochondria during sexual reproduction, creating progeny that inherits cytoplasmic genomes solely from either the maternal or the paternal side (Birky 1995).

The phenomenon of uniparental inheritance was first discovered over a century ago. Correns documented the case in *Mirabilis jalapa*, which was later identified as maternal inheritance of plastid genomes. In the same year, Baur reported non-Mendelian inheritance of green-white variegation in *Pelargonium zonale*, which was later attributed to biparental plastid inheritance (Baur 1908; Correns 1908; Hagemann 2010). These seminal studies demonstrated that the vertical transmission pattern of cytoplasmic genomes varies across species. Subsequent research has suggested that maternal inheritance of plastids and mitochondria is the predominant transmission pattern in most species (Greiner et al. 2015; Schneider 2023).

**Figure 2.**
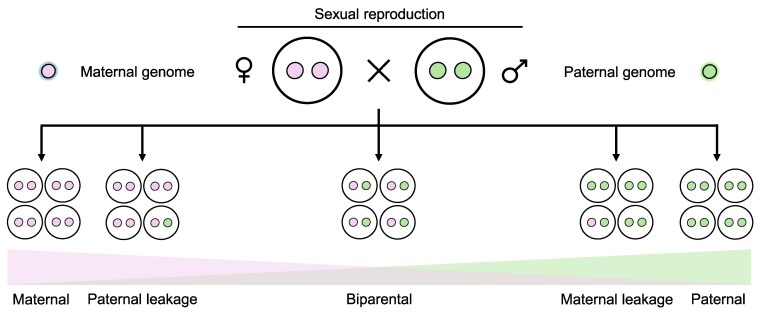
Different species exhibit various vertical transmission patterns of cytoplasmic genomes. While strict uniparental inheritance was once considered the standard, recent findings suggest that leakages occur more frequently than previously assumed. Instead of a binary classification (uniparental vs. biparental), transmission patterns are represented as a spectrum based on the frequency distribution of parental genomes detected in the progeny.

Recent findings have provided deeper insights into the vertical transmission of cytoplasmic genomes, revealing that the binary classification of uniparental versus biparental inheritance is overly simplistic. Instead, a wide spectrum of transmission patterns has been observed, including maternal; paternal; maternal inheritance with paternal leakage; paternal inheritance with maternal leakage; and biparental inheritance (Fig. 2) (Birky 1995; Camus et al. 2022). Apparently, no universal mechanism dictates the vertical transmission of cytoplasmic genomes. Instead, different species utilize diverse cellular processes to shape their inheritance patterns. These dynamic and variable mechanisms of vertical transmission differ significantly from the Mendelian inheritance of the nuclear genome (Birky 1995; Mogensen 1996).

## The interplay between vegetative segregation and uniparental inheritance

**Figure 3.**
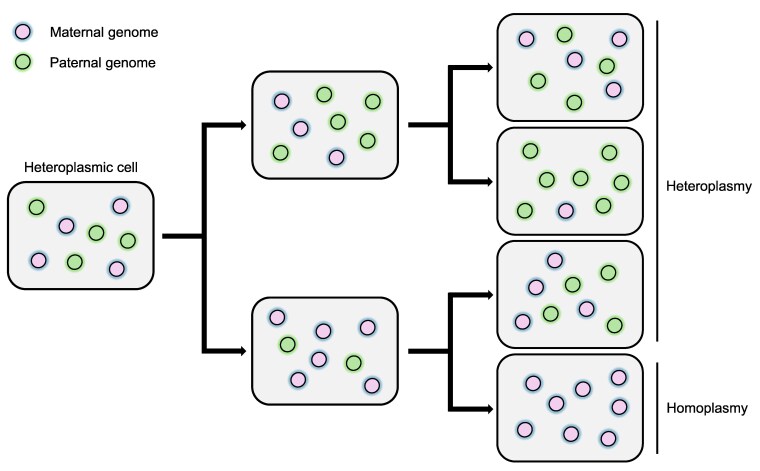
An illustration depicting the segregation of parental cytoplasmic genomes across successive cell division cycles. Assuming relaxed replication and random partitioning of parental genomes, their distribution among daughter cells follows a stochastic pattern. This leads to fluctuations in heteroplasmy levels over time, either increasing or decreasing, and eventually results in the formation of homoplasmic daughter cells (bottom right).

Although maternal inheritance of cytoplasmic genomes is predominant in most species, its enforcement is not always absolute. Notably, progeny from interspecific crosses often exhibit a higher rate of paternal leakage (Xu 2005; Shrestha et al. 2021). Occasional paternal leakage leads to the formation of heteroplasmic zygotes containing both paternal and maternal cytoplasmic genomes. Nevertheless, heteroplasmy is typically resolved through vegetative segregation in subsequent cell divisions. Over time, daughter cells derived from heteroplasmic zygotes retain only paternal or maternal genomes, ultimately achieving a homoplasmic state (Fig. 3) (Birky 2001).

**Figure 4.**
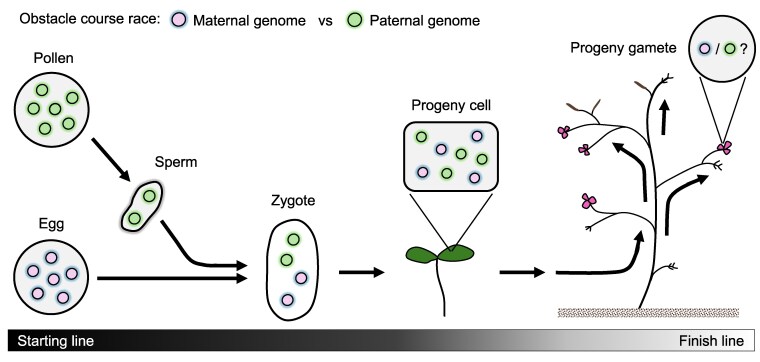
An illustration of an obstacle course race between paternal and maternal cytoplasmic genomes, tracing their path from parental to progeny gametes. This journey repeats to ensure the inheritance of cytoplasmic genomes across generations.

The relentless interplay of vegetative segregation and uniparental inheritance drives the system toward homoplasmy. Consequently, either the paternal or maternal cytoplasmic genomes are preserved and transmitted through generations. This dynamic can be envisioned as a competition between parental cytoplasmic genomes, wherein one eventually becomes the dominant variant within the population. To illustrate this competition, I propose an analogy of an obstacle course race (Fig. 4).

The starting lines are set in the male and female gametes, from which the parental cytoplasmic genomes must overcome various obstacles to reach the zygote upon fertilization. Following embryogenesis and development, the genomes that successfully persist and reach the progeny gametes emerge as the winners, securing their transmission to future generations. In the following sections, I will expand on this “obstacle course race” analogy and outline the obstacles encountered by paternal and maternal cytoplasmic genomes along the course.

## Obstacle course race: paternal vs maternal cytoplasmic genomes

### The starting line—parental gametes set off

Paternal and maternal genomes embark on this obstacle course race from the male and female gametes, respectively. As gametes act as vehicles that transport DNA to the next generation, an effective way to regulate the transmission of cytoplasmic genomes is to control their abundance within gametes. In an extreme scenario, if sperm cells entirely lack cytoplasmic genomes while egg cells retain them, strict maternal inheritance would occur, or vice versa. Even in less extreme cases, substantial differences in cytoplasmic genome abundance between male and female gametes can significantly affect the inheritance pattern. Therefore, cellular processes that regulate cytoplasmic genome abundance in gametes play an important role in shaping inheritance patterns.

#### Exclusion of plastids and mitochondria from gametes

Anisogamous reproduction is common in plants, where parents produce gametes of different sizes (Bell 1978). Sperm cells are considerably smaller than egg cells, which limits the number of plastids and mitochondria that can be transmitted from the paternal side.

**Figure 5.**
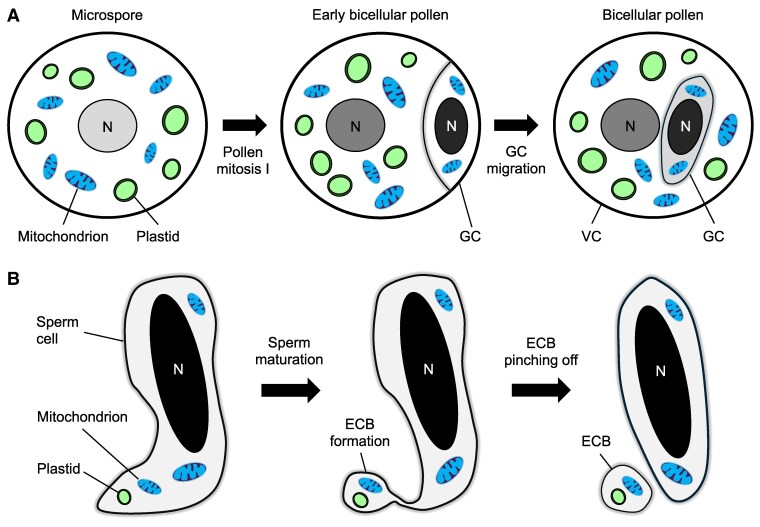
Organelle exclusion and disposal mechanisms in maturing pollen and sperm cells. **A)** During pollen mitosis I, an asymmetric cell division gives rise to a small generative cell within early bicellular pollen. At this stage, organelle exclusion results in the exclusion of plastids and most mitochondria from the newly formed generative cell. Consequently, the generative cell contains only a limited number of organelles, restricting their transmission through the male gametes. **B)** During sperm maturation, the enucleated cytoplasmic body (ECB) composed of cytoplasmic projections enclosing plastids and mitochondria, forms at the cell surface. Through vesiculation and subsequent pinching off, the ECB detaches from the cell body, facilitating the removal of organelles in maturing sperms. N, nucleus; VC, vegetative cell; GC, generative cell.

The reduced sperm size results from an asymmetric cell division during male gametogenesis: At pollen mitosis I, the microspore divides asymmetrically, producing a bicellular pollen with a large vegetative cell and a small generative cell. The generative cell then undergoes pollen mitosis II, giving rise to 2 sperm cells. It is at the time of pollen mitosis I, that plastids and mitochondria are largely excluded from the generative cell. In most plant species, plastids are rarely observed in the generative cell (Sears 1980; Hagemann and Schröder 1989), while mitochondria are still present but in limited numbers (Mogensen 1996). This physical exclusion of organelles serves as a barrier against the transmission of paternal genomes and is recognized as a key mechanism underlying maternal inheritance (Fig. 5A) (Birky 1995). However, important questions remain: is there an active mechanism that deliberately excludes plastids and mitochondria, or is this exclusion process merely passive, resulting from the limited size of the generative cell?

Cytological studies have been performed to explore the organelle exclusion mechanism, with a focus on investigating plastid distribution during pollen development. Prior to pollen mitosis I, plastids are randomly distributed throughout the microspore cytoplasm. However, during prophase, plastids become polarized and cluster at the pole opposite to the site where the generative cell will form (Hagemann and Schröder 1989). It is speculated that this polarization and clustering of plastids are mediated by the actin cytoskeleton (Schroder et al. 1988), microtubules (Van Went 1984; Brown and Lemmon 1991; Tanaka 1991), and the endoplasmic reticulum (Bohdanowicz and Lewandowska 1999). However, direct evidence demonstrating these cellular components modulating plastid distribution during pollen mitosis I remains lacking.

On the other hand, generative cell size has been shown as a key factor influencing organelle abundance in male gametes. The small volume of the generative cell inherently restricts its ability to house organelles, thereby severely limiting the number of organelles transmitted by the male gamete. In *Gasteria verrucosa* and *Tulbaghia violacea* pollen, plastids are typically absent from the small generative cell. However, in atypical pollen grains with larger generative cells, the exclusion of plastids is compromised (Schröder 1985; Schröder and Oldenburg 1990). A recent study in *Nicotiana tabacum* revealed that low temperatures disrupt asymmetric cell division during pollen mitosis I, resulting in a higher frequency of plastids being included in the generative cell (Chung et al. 2023).

It is possible that both (1) organelle polarization and clustering, and (2) the small size of the generative cell, contribute to the exclusion of organelles from the male gamete. However, the exclusion mechanisms appear to be imperfect, particularly in the case of mitochondria. It is also important to note that the gamete size hypothesis cannot account for the uniparental inheritance patterns observed in algae that produce isogamous gametes (Sager and Lane 1972). Additional factors, beyond gamete size, regulate the vertical transmission of cytoplasmic genomes.

Notably, a size-independent mechanism for excluding organelles from sperm cells has been identified. This process involves the formation of enucleated cytoplasmic bodies (ECBs), which encapsulate cytoplasmic organelles and are eventually pinched off from the sperm cells (Fig. 5B) (Mogensen 1996). Early electron microscopy studies on *Hordeum vulgare* pollen provided evidence for this organelle disposal mechanism, showing that mitochondrial abundance is halved during sperm cell maturation (Mogensen and Rusche 1985). In addition, sperm cell-derived ECBs have also been reported in *N. tabacum*. ECBs form at the cytoplasmic projection at the trailing end of the sperm cell (Yu et al. 1992). However, the molecular mechanisms underlying ECB formation and whether an active process sequesters organelles into ECBs remain unknown. Once separated from the cell body, the cellular contents of the ECBs degenerate (Yu et al. 1992). The elimination of plastids and mitochondria via ECBs further prevents the transmission of paternal genomes.

Compared to male gametes, considerably less is known about the distribution and dynamics of plastids and mitochondria during female gametogenesis. In most angiosperms, female gametes typically contain numerous plastids, with the notable exception of *Quercus gambelii*, where plastids are unusually scarce in egg cells (Mogensen 1972). Intriguingly, research on various gymnosperm species has revealed an almost complete absence of plastids in egg cells, likely explaining the predominantly paternal plastid inheritance in these species (Hagemann 1992). The unequal distribution of plastids during female gametophyte development may contribute to their absence in egg cells, although the mechanisms behind this process are still unclear.

#### Degradation of plastids and mitochondria in gametes

**Figure 6.**
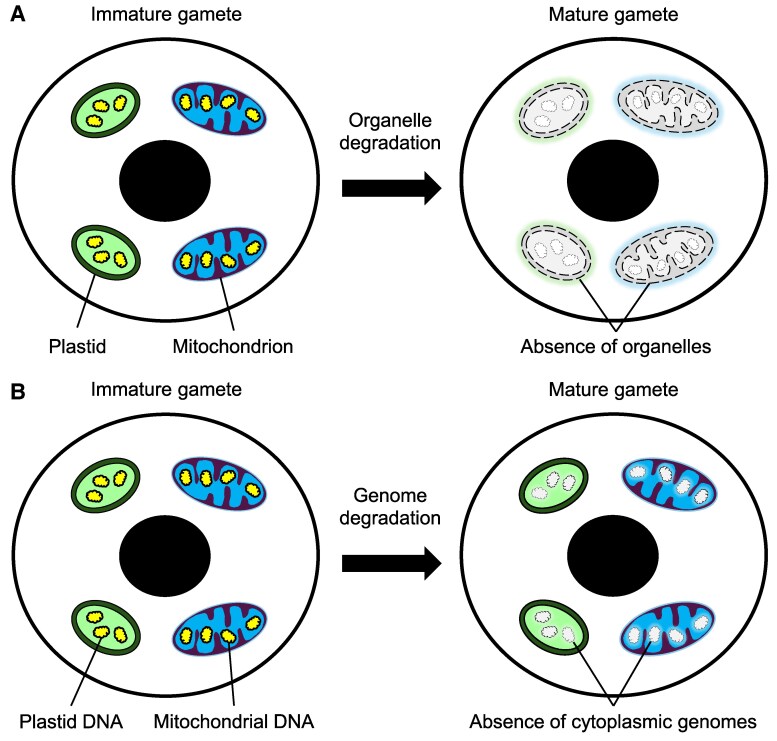
Schematic diagrams illustrating the mechanisms of organelle degradation and genome degradation. During gamete maturation, these degradation processes facilitate **A)** the removal of entire organelles; and **B)** the elimination of cytoplasmic genomes, thereby preventing their vertical transmission.

In addition to organelle exclusion and disposal mechanisms, organelle degradation also reduces plastid and mitochondrial abundance in gametes (Fig. 6A). Electron microscopy has revealed signs of plastid degradation, such as the disintegration of membrane structures and swelling of plastids (Vaughn et al. 1980). The timing of degradation varies across species. Previous studies have demonstrated that plastid degradation takes place in generative cells (Clauhs and Grun 1977; Schröder 1986; Bednarska 1988; Pacini et al. 1992; Yu and Russell 1994). In some species, however, this process occurs later in sperm cells (Schmitz and Kowallik 1987; Primavesi et al. 2017). While plastid degradation is well-documented in male gametes, reports of similar processes in female gametes are far less common. An exceptional example is reported in *Daucus muricatus*, where degenerated plastids were observed at the periphery of the egg cell (Hause 1991).

Direct evidence for mitochondrial degradation in gametes is scarce. In Nicotiana tabacum, mitochondria have been observed within autophagic vacuoles in generative cells, suggesting an autophagy-mediated degradation process (Yu and Russell 1994). In other species, morphological changes in mitochondria and alterations in the cristae structure have been reported (Mogensen and Rusche 1985; Wang et al. 2010; Liu 2012). However, these changes should be interpreted cautiously, as they may reflect shifts in physiological states rather than definitive signs of degradation. Nonetheless, organelle degradation represents an alternative strategy to facilitate the progressive elimination of plastids and mitochondria in gametes, thereby governing the vertical transmission of cytoplasmic genomes.

#### Degradation of cytoplasmic genomes in gametes

Instead of degrading the entire organelle, a more refined approach involving the selective degradation of cytoplasmic genomes can also significantly influence the inheritance pattern (Fig. 6B). In most angiosperms, cytoplasmic genomes are present in young pollen but are either absent or found in very low amounts in mature pollen (Miyamura et al. 1987; Corriveau and Coleman 1988, 1991; Zhang et al. 2003; Wang et al. 2010). An active genome degradation process has been shown to occur during pollen maturation, leading to the elimination of paternal cytoplasmic genomes and ensuring maternal inheritance (Kuroiwa 2010; Sakamoto and Takami 2024).

Efforts have been made to identify the nucleases responsible for the degradation of cytoplasmic genomes in maturing pollen (Nakamura et al. 1992; Sodmergen et al. 1992). Forward genetic screens in *Arabidopsis thaliana* led to the discovery of the exonuclease DEFECTIVE IN POLLEN ORGANELLE DNA DEGRADATION1 (Matsushima et al. 2011) (Box 1). In Zea Mays, a 20-kD endonuclease (M20) has been identified in pollen mitochondria. Its Arabidopsis homolog, AtM20, degrades mitochondrial genomes but not plastid genomes in pollen (Ma et al. 2018). The role of M20 in regulating mitochondrial inheritance remains unclear. Nonetheless, the identification of mitochondria-specific nucleases presents an intriguing possibility: mitochondrial genomes are selectively degraded while plastid genomes are preserved in pollen, leading to maternal mitochondrial inheritance while allowing potential paternal plastid transmission. Several species have been reported where either plastid or mitochondrial genomes undergo selective degradation (Nagata et al. 1999; Ji et al. 2004; Liu et al. 2004). Investigating the mechanisms underlying this phenomenon and examining its correlation with vertical transmission patterns would be highly intriguing.

Box 1. **DPD1, a long-sought nuclease that governs plastid inheritance**

DPD1, initially characterized in Arabidopsis thaliana, is an exonuclease that degrades both plastid and mitochondrial genomes (Matsushima et al. 2011). Its expression is tissue-specific, with high levels in senescing leaves and maturing pollen (Tang and Sakamoto 2011). In vegetative tissues, AtDPD1 facilitates efficient phosphate utilization and recycling by breaking down cytoplasmic genomes (Takami et al. 2018). In maturing pollen, AtDPD1 degrades cytoplasmic genomes and was speculated to ensure maternal inheritance. However, the original study lacked sufficient experimental scale to conclusively confirm its role in regulating cytoplasmic inheritance (Matsushima et al. 2011; Sakamoto and Takami 2024).

A recent large-scale study in Nicotiana tabacum revealed the role of DPD1 in plastid inheritance. In Ntdpd1 mutants, plastid genomes are retained in mature pollen, leading to a significant increase in paternal plastid transmission when used as pollen donors (Chung et al. 2023). This study highlights DPD1 as a key regulator of plastid inheritance and presents compelling evidence for the role of the genome degradation mechanism in this process.

Does DPD1 regulate cytoplasmic inheritance in other species?

In seed plants, DPD1 is conserved and functions as a standalone exonuclease (Takami et al. 2018). Notably, a recent study identified DPD1-like exonucleases fused to DNA-binding proteins, such as the helicase RecG and MutS-like proteins, in land plants and green algae (Sloan et al. 2024). While the function of these DPD1-fused proteins remains to be explored, the role of DPD1 in cytoplasmic genome degradation has been reported in crops. In Oryza sativa, 2 DPD1 homologs have been identified that participate in cytoplasmic genome degradation, though their role in cytoplasmic inheritance remains unexamined (Islam et al. 2024). In *Cucumis sativus*, a homolog termed CsDPD1 has been characterized. CsDPD1 functions similarly to AtDPD1, as its expression in Atdpd1 mutants rescues the genome retention phenotype in mature pollen (Shen et al. 2015). Despite high CsDPD1 expression in cucumber pollen, substantial mitochondrial DNA persists in generative cells, potentially explaining the unique paternal mitochondrial inheritance observed in cucumbers (Havey 1997; Munasinghe and Ågren 2023). Notably, these findings suggest the involvement of additional factors that may protect mitochondrial genomes from DPD1-mediated degradation. In Arabidopsis, it has been shown that the overexpression of the mitochondrial DNA-binding protein WHIRLY2 results in the retention of the mitochondrial genome in mature pollen (Cai et al. 2015). Whether WHIRLY2 affects mitochondrial inheritance remains unclear. However, these findings indicate that other proteins also contribute to the maintenance and degradation of cytoplasmic genomes in pollen, potentially playing key roles in shaping inheritance patterns.

### Syngamy hurdles—the journey from gametes to zygotes

Various mechanisms operating in gametes can impede the vertical transmission of cytoplasmic genomes. Even if cytoplasmic genomes successfully overcome these obstacles, additional challenges that arise during syngamy may still prevent their transmission to the zygote. Here, I will highlight the cellular processes during fertilization that profoundly impact the cytoplasmic inheritance patterns.

#### Cytoplasmic stripping

One remarkable process, known as cytoplasmic stripping, prevents paternal inheritance by excluding sperm cytoplasm from the egg during syngamy. While the sperm nucleus enters the egg upon gamete fusion, paternal plastids and mitochondria remain in the cytoplasmic bodies outside of the egg. Consequently, paternal cytoplasmic genomes are stripped away along with the sperm cytoplasm (Sears 1980; Hagemann and Schröder 1989; Mogensen 1996). Cytoplasmic stripping has been observed in cotton (Jensen and Fisher 1967), spinach (Wilms 1981), barley (Mogensen 1988), poplar (Russell et al. 1990), and lily (Janson 1992), facilitating maternal inheritance in these species.

#### Sperm dimorphism and preferential fertilization

**Figure 7.**
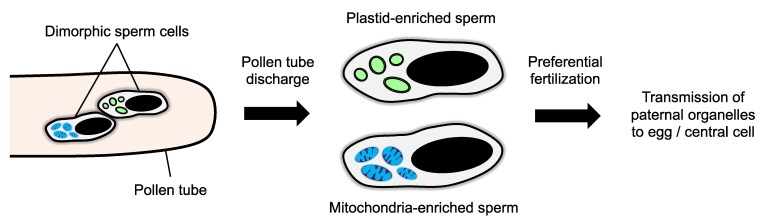
An example of sperm cytoplasmic dimorphism. Two sperm cells with distinct cytoplasmic contents are delivered to the female gametes via the pollen tube. Upon pollen tube discharge, 1 sperm cell may preferentially fuse with the egg, transmitting its organelles to the resulting zygote.

Another intriguing process, involving sperm dimorphism followed by preferential fertilization, regulates the contribution of paternal organelles to the zygote (Fig. 7) (Russell 1987; Mogensen 1996).

Following pollen mitosis II, a pair of sperm cells is formed: one fuses with the egg cell to form the zygote, while the other fuses with the central cell to form the endosperm. While it is widely assumed that the 2 sperm cells are isomorphic, accumulating evidence suggests that sperm dimorphism may be more prevalent than previously thought (Mogensen 1992; Tian et al. 2001; Weterings and Russell 2004; Yang et al. 2005). Dimorphic sperm cells can differ in volume, cellular contents, or organelle composition (Russell 1991). Several mechanisms have been proposed to explain the emergence of sperm dimorphism, including the polarized distribution of organelles during pollen mitosis II and the differential formation of ECBs from specific sperm cells (Tian et al. 2001; Saito et al. 2002). A notable example is reported in *Plumbago zeylanica*, where the 2 sperm cells exhibit cytoplasmic dimorphism: one is enriched with plastids, while the other contains numerous mitochondria but lacks plastids (Russell 1984). Notably, the plastid-enriched sperm cell preferentially fuses with the egg cell (Russell 1985), enabling the transmission of paternal plastids to zygotes. These findings underscore the significance of sperm dimorphism and preferential fertilization in regulating cytoplasmic inheritance (Saito et al. 2001; Hagemann 2004).

### Zygotic crossroads—diverging fates of parental genomes

After navigating the first part of the obstacle course race, parental plastids and mitochondria successfully reach the zygote. However, their mere presence in the zygote does not guarantee their smooth journey to the finish line, as cellular processes in the developing zygote will determine their fate.

#### Selective degradation of cytoplasmic genomes in zygotes

**Figure 8.**
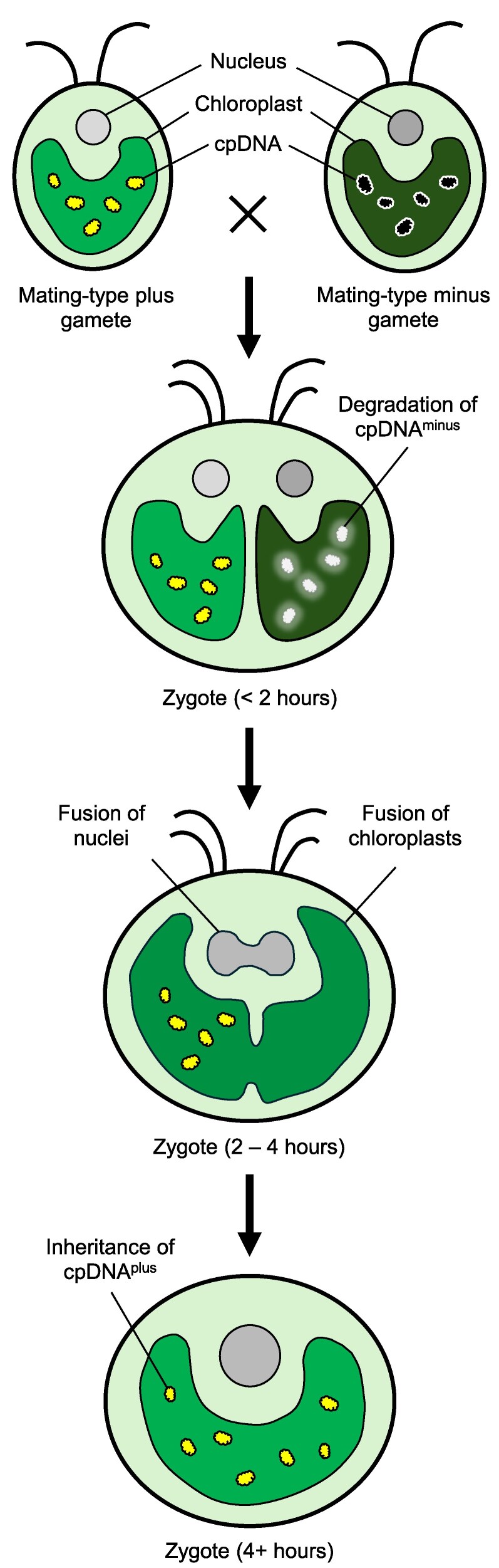
A schematic diagram illustrating the selective degradation of chloroplast genomes in Chlamydomonas zygotes. During sexual reproduction, an MT-plus and an MT-minus gamete pair up and fuse to form a binucleate zygote. At the early stage after gametic fusion, the MT-minus chloroplast DNA (cpDNA⁻) undergoes selective degradation. Between 2 and 4 h post-fusion, the chloroplasts and nuclei of MT-plus and MT-minus gametes merge. By 4 h, the zygote contains a single chloroplast carrying only the MT-plus chloroplast genome (cpDNA⁺), resulting in the uniparental inheritance of cpDNA.

Genome degradation appears to be a widely adopted mechanism for controlling cytoplasmic inheritance. Even when cytoplasmic genomes escape degradation in gametes, additional processes in the zygote may selectively eliminate either paternal or maternal genomes. A well-studied example of this occurs in the green alga *Chlamydomonas reinhardtii* (Fig. 8).

In Chlamydomonas, the gametic sex is determined by 2 mating type (MT) loci: MT-plus and MT-minus. During reproduction, an MT-plus gamete and an MT-minus gamete fuse to form a zygote that initially contains chloroplasts and mitochondria from both parents (Goodenough 2023). Within 40 to 50 min of gamete fusion, the chloroplast genomes from the MT-minus gamete (cpDNA^minus^) are selectively degraded, leading to the uniparental inheritance of MT-plus chloroplast genomes (cpDNA^plus^) (Kuroiwa et al. 1982; Nishimura et al. 1999). Intriguingly, the opposite occurs with mitochondrial genomes: the MT-plus mitochondrial DNA (mtDNA^plus^) is degraded during zygote maturation, resulting in the exclusive transmission of the MT-minus mitochondrial genome (mtDNA^minus^) (Boynton et al. 1987; Beckers et al. 1991). These selective genome degradation mechanisms ensure uniparental inheritance in Chlamydomonas.

Since the discovery of these mechanisms, extensive efforts have been made to identify the genes involved (Sager and Ramanis 1973; Nakamura 2010; Nishimura 2010; Joo et al. 2023). A Ca²⁺-dependent nuclease, desginated as MT-plus-specific DNase (MDN), has been proposed as a candidate for the preferential digestion of cpDNA^minus^ in zygotes (Nishimura et al. 2002). Although MDN is expressed in the MT-plus gamete, cpDNA^plus^ remains protected from MDN activity. Upon gamete fusion, MDN gains access to the MT-minus chloroplast and degrades the unprotected cpDNA^minus^, leading to the uniparental inheritance of cpDNA^plus^ (Nishimura 2010).

In contrast to the rapid degradation of chloroplast genomes, the elimination of mtDNA^plus^ is a slow and progressive process (Nakamura 2016). mtDNA^plus^ is still detected 6 h after mating, with gradual degradation occurring during zygote maturation. Interestingly, complete elimination of mtDNA^plus^ requires light exposure, indicating a markedly different degradation mechanism compared to that of chloroplast genomes (Aoyama et al. 2006).

To date, the exact nucleases and molecular mechanisms governing cytoplasmic inheritance in Chlamydomonas have not been identified. Nevertheless, several mutants exhibiting defects in selective genome degradation and altered inheritance patterns have been isolated (Gillham et al. 1987; VanWinkle-Swift et al. 1994; Armbrust et al. 1995; Joo et al. 2022) (Box 2). In addition, genetic screens have identified GSM1 and GSP1 as key players regulating the zygotic developmental program and uniparental inheritance (Nishimura et al. 2012; Kariyawasam et al. 2019). During mating, GSM1 and GSP1 physically interact to form a heterodimer and regulate the expression of early zygote-specific genes (Lee et al. 2008), subsequently driving the selective degradation of cpDNA^minus^ and mtDNA^plus^ in Chlamydomonas zygotes (Nishimura et al. 2012).

Box 2. **Otu2p, a protector of the MT-plus chloroplast genomes**

Instead of identifying the long-sought nucleases, Joo et al. discovered an otubain-like deubiquitinase (Otu2p) that acts as a chloroplast genome protector, preserving the integrity of cpDNA^plus^ and ensuring their uniparental inheritance in Chlamydomonas (Joo et al. 2022).

Encoded within the sex-determining MT-plus locus, Otu2p expression prevents the proteasome-mediated degradation of the preprotein translocase of the chloroplast outer membrane (TOC) in MT-plus gametes (Joo et al. 2022). TOC complex components are essential for the efficient preprotein import into the chloroplasts (Ling et al. 2012). The maintenance of functional TOC complex in MT-plus gametes creates an asymmetry in protein import capacity between the MT-plus and MT-minus chloroplasts. This asymmetry biases the import of DNA repair proteins toward the MT-plus chloroplast, promoting the maintenance of cpDNA^plus^. In contrast, the limited import capacity of MT-minus chloroplasts hampers genome maintenance, leading to the accumulation of damaged DNA and its subsequent degradation. This sex-linked organelle quality control mechanism selectively preserves cpDNA^plus^ integrity, leading to the uniparental inheritance of high-quality chloroplast genomes (Joo et al. 2022).

This study offers several intriguing insights. First, it uncovers a ubiquitin-dependent mechanism that differentiates parental chloroplasts. A similar system has been reported in animals, which the paternal mitochondria are selectively labeled and degraded by the ubiquitin-proteasome system (Sutovsky et al. 1999; Politi et al. 2014; Song et al. 2016). Second, this study provides evidence supporting previous speculations that the fate of parental chloroplast genomes is not strictly predetermined. If the quality or abundance of cpDNA^plus^ is compromised, the degradation of cpDNA^minus^ is delayed (Wurtz et al. 1977; Armbrust et al. 1995). This compensatory mechanism ensures that the zygote can sustain an adequate level of chloroplast genomes to support its maturation and development (Joo et al. 2022).

In summary, the Otu2p-mediated mechanism offers new insights into chloroplast inheritance. More importantly, it illustrates how uniparental inheritance can occur in isogamous gametes and explains how this inheritance is achieved even when both parental genomes are contributed to the zygote.

#### Preferential replication of cytoplasmic genomes

Preferential genome replication determines the fate of parental genomes by affecting their relative abundance in zygotes. In C. reinhardtii, differences in the methylation status between cpDNA^plus^ and cpDNA^minus^ result in varying genome replication rates (Umen and Goodenough 2001; Nishiyama et al. 2004). Extensive methylation in cpDNA^plus^ has been observed in gametes and zygotes (Burton et al. 1979; Royer and Sager 1979; Lopez et al. 2015). Upon zygote germination, the hypermethylated cpDNA^plus^ confers a replication advantage, contributing to its dominance and uniparental inheritance (Umen and Goodenough 2001). When MT-plus gametes are treated with 5-azacytidine, a drug that induces hypomethylation of cpDNA^plus^, this replication advantage is diminished. In this case, the relative replication rate of cpDNA^minus^ increases, leading to the breakdown of uniparental inheritance (Umen and Goodenough 2001).

Beyond methylation status, genome quality also affects replication efficiency and, consequently, inheritance patterns. Treatment of Chlamydomonas MT-plus gametes with the DNA-damaging agent methanesulfonic acid ethyl ester (EMS) reduces their genome quality, disrupting the uniparental inheritance of cpDNA^plus^ (Umen and Goodenough 2001). Together, selective methylation and preferential genome replication enable the differentiation of parental genomes and genome quality, providing a sophisticated mechanism for regulating cytoplasmic inheritance.

#### Positioning of plastids and mitochondria in zygotes

While mechanisms controlling genome abundance and quality are essential, the spatial positioning of plastids and mitochondria within the zygote also significantly influences the fate of cytoplasmic genomes.

**Figure 9.**
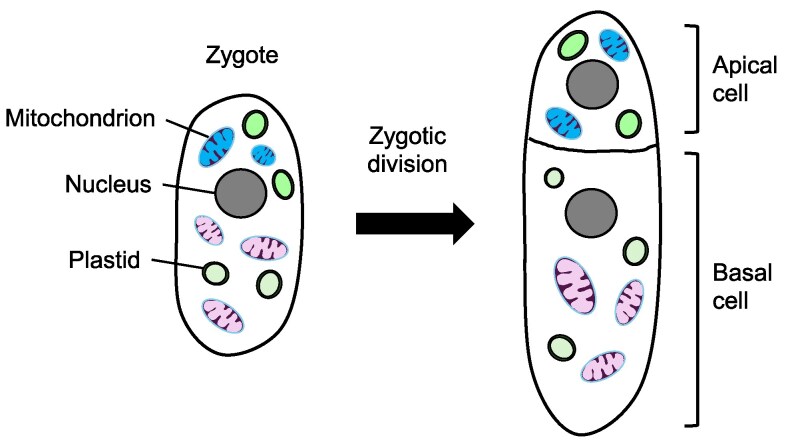
An example of the position effect observed in angiosperm zygotes. A small apical cell and a large basal cell are formed upon zygotic division. Only the plastids and mitochondria allocated to the apical cell retain the potential for transmission into the shoot tissues of the progeny plant.

In angiosperms, zygote division is typically asymmetric, producing a small apical cell and a large basal cell. Organelles that are partitioned into the apical cell are inherited, as this cell forms the embryo lineage generating the majority of the plant body. In contrast, the basal cell develops into the suspensor and hypophysis, structures that do not contribute to the embryo proper and represent a “dead end” for cytoplasmic genomes (Fig. 9) (Mogensen 1996).

Previous research has shown that the positioning of the plastid and mitochondrion dictates their allocation to either the apical or basal cells. This position-dependent effect is reported in Medicago sativa. Here, maternal plastids positioned below the midsection of the egg nucleus are incorporated into the basal cell upon zygotic division. As the basal cell does not contribute to the embryo, these maternal plastids are not further inherited (Zhu et al. 1993; Rusche et al. 1995). In gymnosperms, organelles concentrated in the perinuclear zone around fusing gamete nuclei are more likely to be inherited by the embryo (Whatley 1982; Hagemann 1992; Mogensen 1996). In Biota orientalis, paternal plastids and mitochondria cluster around the zygote nucleus, leading to the paternal inheritance of cytoplasmic genomes (Chesnoy 1977). Conversely, in Ephedra distachya, maternal organelles cluster around the perinuclear zone while paternal organelles remain at the micropylar entry point (Moussel 1978). This spatial arrangement enforces maternal inheritance, making Ephedra an exception among gymnosperms, where paternal inheritance is typically observed. Organelle positioning also explains contrasting inheritance patterns in Pseudotsuga menziesii (Neale et al. 1986; Marshall and Neale 1992). In eggs, mitochondria form a dense cluster around the nucleus, whereas plastids are less concentrated in this region. During fertilization, paternal organelles accompany the sperm nucleus as it migrates toward the egg nucleus. Consequently, the perinuclear zone is enriched with maternal mitochondria and paternal plastids, leading to their selective inheritance in the proembryo (Owens and Morris 1990, 1991). These examples highlight the importance of organelle positioning in inheritance. Cytoskeleton has been shown to facilitate organelle movement and polar nuclear migration (Sheahan et al. 2004; Kimata et al. 2016). Notably, actin cables are crucial for forming the filamentous structure of zygotic mitochondria in Arabidopsis (Kimata et al. 2020). These findings highlight the role of the actin cytoskeleton in mitochondrial fusion, fission, and dynamic positioning in maturing zygotes. The precise function of the cytoskeleton in organelle positioning during zygotic division warrants further investigation.

### Embryogenesis and beyond—relentless segregation of cytoplasmic genomes

After zygotic division, the apical cell may still contain both paternal and maternal cytoplasmic genomes. Heteroplasmy persists, with the race between parental genomes continuing until homoplasmy is established. During embryogenesis and plant development, vegetative segregation enforces homoplasmy through repeated cell divisions. Since only a finite number of plastids and mitochondria are transmitted from mother to daughter cells, cells gradually acquire a uniform set of cytoplasmic genomes and become homoplasmic (Birky 2001). This process creates a within-individual drift, which has profound implications for the transmission of cytoplasmic genomes into germ cells and future generations. Mathematical models have been developed to simulate the segregation process, highlighting the power of stochastic partitioning (Michaelis 1967; Birky et al. 1983; Broz et al. 2024). Nevertheless, additional factors beyond randomness also influence both the direction and rate of segregation (Zhang et al. 2018; Camus et al. 2022).

#### Steering the segregation direction

Relaxed replication and stochastic partitioning drive the segregation of heteroplasmic genomes through random drift (Birky 2001). However, recent findings suggest that cytoplasmic genome variants, particularly those with phenotypic consequences, deviate from this random segregation pattern (Haig 2016; Van den Ameele et al. 2020; Parakatselaki and Ladoukakis 2021).

In heteroplasmic cells containing both functional and defective genomes, it has been shown that defective genomes are eliminated through purifying selection, while functional genomes are proliferated (Fan et al. 2008; Rajasimha et al. 2008; Stewart et al. 2008; Freyer et al. 2012; Hill et al. 2014; Ma et al. 2014; Palozzi et al. 2018). However, in some cases, genomes carrying deleterious mutations are not only maintained but may even become the dominant population (Ma and O'Farrell 2016; Stewart and Chinnery 2021; Yang et al. 2022). For example, mitochondrial genomes with mutations have been observed to proliferate despite their harmful effects (Tsang and Lemire 2002; Phillips et al. 2015; Milot et al. 2017). These findings indicate that cytoplasmic genomes can experience both positive and negative selection, influencing their relative abundance and heteroplasmy levels. The selection of cytoplasmic genomes operates at multiple levels, ranging from the genome and organelle to the cellular scales, ultimately steering the segregation direction.

#### At the genome level

Cytoplasmic genome variants with higher replication efficiency gain a selective advantage by attaining greater copy numbers, potentially outcompeting other genomes and increasing their likelihood of being transmitted to daughter cells during cell division (Skibinski et al. 1994; Zouros et al. 1994).

A study on an unusual Chlamydomonas strain has offered valuable insights into chloroplast genome replication. This unique heteroplasmic strain, spa19, harbors 2 distinct chloroplast genomes (PS + and PS-). The replication of PS + and PS- genomes is differentially regulated based on the developmental status of the algae. During the vegetative stage, both PS + and PS- genomes are stably maintained, albeit through different replication mechanisms. While PS- genome can be replicated via transcription-, recombination-, or primase-mediated priming, PS + genome is maintained exclusively by transcription-mediated priming. Notably, during the reproductive stage, chloroplast transcription activity is drastically reduced, leading to the suppression of PS + genome replication and the eventual loss of this variant (Nishimura and Stern 2010). This demonstrates how changes in developmental stages and cellular status can impact the replication efficiency of a genome variant, thereby influencing the segregation direction.

In plants, mitochondrial genomes are highly dynamic and exist in various configurations, with one configuration typically being predominant while others are present at sublimon levels (Kmiec et al. 2006; Gualberto et al. 2014). Interestingly, these sublimons can occasionally be selectively amplified through a process called substoichiometric shifting (SSS), leading to changes in the relative abundance of genome variants and altering the segregation dynamics (Woloszynska 2010). Genes that influence the frequency of SSS such as OSB1, MSH1, and RECA3 have been identified (Zaegel et al. 2006; Shedge et al. 2007). However, the mechanism underlying the selective replication of a particular mitochondrial genome variant remains unclear.

#### At the organelle level

The quality of plastids and mitochondria is determined by the genomes they contain. Organelles carrying mutated genomes may become dysfunctional and are removed via organelle quality control mechanisms (Woodson 2016; Ng et al. 2021). In turn, these mechanisms facilitate the selection of cytoplasmic genomes by preserving functional organelles and eliminating defective ones. Intriguingly, since mitochondria undergo active fusion and fission while plastids do not (Sears 1980; Arimura et al. 2004), the quality control of organelles operates through distinct mechanisms.

Mitochondria harboring mutated genomes exhibit defects in the mitochondrial oxidative phosphorylation system, leading to reduced ATP production and altered membrane potential (Twig et al. 2008; Koopman et al. 2013). In Drosophila, these defective mitochondria are isolated by restricted fusion and increased fragmentation, and are subsequently degraded through mitophagy (Lieber et al. 2019). These observations suggest that mitochondrial fusion/fission dynamics facilitate the sequestration and removal of damaged components. In addition, research in animals and yeast has identified proteins involved in autophagy and mitochondrial fusion/fission dynamics as crucial factors in facilitating the elimination of dysfunctional mitochondria carrying mutated genomes (Pickles et al. 2018; Aretz et al. 2020; Zakirova et al. 2021; Spinazzola et al. 2024).

In plants, growing evidence highlights the important role of mitophagy in maintaining mitochondrial quality (Broda et al. 2018; Nakamura et al. 2021 a, 2021b; Duckney et al. 2024). Various mutants with impaired mitophagy show an accumulation of depolarized mitochondria (Dündar et al. 2020; Nakamura et al. 2021a, 2021b; Li et al. 2022). Notably, defects in mitochondrial dynamics result in the formation of clustered mitochondria and hinder the removal of depolarized mitochondria via mitophagy (El Zawily et al. 2014; Ma et al. 2021). These findings indicate a link between mitochondrial dynamics and autophagy-mediated mitochondrial quality control in plants.

Besides mitochondrial quality control, autophagy also plays a role in maintaining plastid quality (Sun et al. 2021). Dysfunctional plastids are selectively eliminated through chlorophagy (Izumi et al. 2017; Nakamura et al. 2018). Intriguingly, autophagy-independent degradation mechanisms also contribute to the removal of damaged chloroplasts (Otegui et al. 2005; Wang and Blumwald 2014; Woodson et al. 2015). The type of chloroplastic stress determines the cellular machinery employed for their removal (Rochaix and Ramundo 2017; Heredia-Martínez et al. 2018; Izumi and Nakamura 2018; Lemke and Woodson 2022), leading to the degradation of either portion of or the entire organelle (Ishida et al. 2008; Michaeli et al. 2014; Zhuang and Jiang 2019; Izumi et al. 2024). Notably, piecemeal chlorophagy involves the formation of vesicular bodies that bud off from plastids, carrying plastid-derived cargos destined for degradation (Otegui 2018). This process resembles the selective removal of mitochondrial subdomains containing mutated genomes in animals (Sen et al. 2022; Tábara et al. 2024). However, no evidence has yet been found to indicate that plastid genomes are selectively removed via piecemeal chlorophagy.

Instead of degradation, differential replication of plastids has been shown to affect the segregation of plastid genomes. In Pelargonium, nuclear Pr genes influence plastid inheritance by selectively regulating plastid replication (Tilney-Bassett 1973). In Oenothera, plastid replication is influenced by the compatibility between the plastid and nuclear genomes, where compatible plastids are capable of efficient replication and are selectively maintained (Chiu and Sears 1993). Recent research has also identified plastid-encoded genes involved in lipid biosynthesis that influence the plastid division rate (Sobanski et al. 2019), highlighting the role of the plastid genome in determining replication efficiency (Chiu et al. 1988). Faster-replicating plastids tend to outcompete slower-replicating ones. Consequently, differences in replication efficiency between parental plastids play a significant role in shaping segregation patterns (Greiner et al. 2015).

#### At the cellular level

Heteroplasmic cells containing deleterious cytoplasmic genomes may not exhibit any phenotypic consequences as long as sufficient functional organelles are present to support essential cellular processes. However, once a critical threshold is surpassed, malfunctioning organelles begin to impair cellular function (Rossignol et al. 2003; Stewart and Chinnery 2015). This can place such cells at a proliferative disadvantage compared to those with functional cytoplasmic genomes. In extreme cases, dysfunctional organelles may trigger signaling pathways that lead to programmed cell death (Ambastha et al. 2015; Van Aken and Van Breusegem 2015; Bock and Tait 2020; Moehlman and Youle 2020; Woodson 2022). These examples illustrate that selection of cytoplasmic genomes can occur at the cellular level, with cells experiencing fitness penalties caused by deleterious cytoplasmic genomes being eliminated. The selection, however, is context-dependent. Cells can accumulate high levels of mutated genomes in conditions where the mutations confer a fitness advantage, highlighting the role of gene–environment interactions in the selection and segregation of cytoplasmic genomes (Vander Heiden et al. 2009; Kotrys et al. 2024).

In plants, dysfunctional plastids and mitochondria have a significant impact on cellular fitness, as these organelles are vital not only for energy production but also for various cellular processes and stress responses (Welchen et al. 2021; Sierra et al. 2023). Heteroplasmic progeny resulting from interspecific crosses may experience cytonuclear incompatibility. This incompatibility disrupts the coordinated cytonuclear interactions, leading to impaired organelle function and reduced cellular fitness (Barnard-Kubow et al. 2017; Postel and Touzet 2020; Postel et al. 2024). For instance, plastid-nuclear incompatibility often manifests as chlorophyl deficiency, leading to chlorosis and variegation phenotypes (Greiner 2012; Barnard-Kubow et al. 2016). In severe cases, such incompatibility can result in cell death (Stubbe 1963). This phenomenon resembles programmed cell death reported in animals, suggesting that plants may employ similar mechanisms to facilitate the segregation of cytoplasmic genomes based on cell fitness.

#### Modulating the segregation rate

Besides the segregation direction, the rate at which heteroplasmy segregation occurs is equally important. Heteroplasmy can be detrimental (Sharpley et al. 2012; Wallace and Chalkia 2013); therefore, efficient segregation and transition to homoplasmy are beneficial. Moreover, a higher segregation rate promotes cell-to-cell variance, facilitating efficient selection of cytoplasmic genomes (Radzvilavicius and Johnston 2022).

In animals, a genetic bottleneck mechanism facilitates the efficient segregation of mitochondrial genomes (Stewart and Chinnery 2015). A special focus has been placed on the bottleneck in the female germline, which accounts for rapid changes in the frequency of mutated mitochondrial genomes across generations (Olivo et al. 1983; Ashley et al. 1989; Jenuth et al. 1996). Notably, the germline bottleneck is proposed to act as a purifying selection mechanism, effectively eliminating deleterious mitochondrial mutations (Bergstrom and Pritchard 1998; Stewart et al. 2008; Edwards et al. 2021). Several mechanisms have been proposed to constitute the germline bottleneck (Stewart and Larsson 2014): (1) a reduction in mitochondrial genome copy number in germ cells (Cree et al. 2008); (2) clustering of identical mitochondrial genomes into homoplasmic nucleoids (Cao et al. 2007; Cao et al. 2009); and (3) selective replication of a random subset of mitochondrial genomes during oocyte maturation (Blok et al. 1997; Wai et al. 2008). These processes lead to dramatic shifts in heteroplasmy levels and may operate in concert to enforce a tight bottleneck in the female germline.

An intriguing question is whether similar mechanisms create a bottleneck in plants. Plant germline development differs markedly from that in animals. While most animals have a developmentally defined, early-segregated germline, plants are generally considered to lack such a feature. However, recent research has challenged this assumption, suggesting that plants may possess a slowly dividing functional germline cell lineage analogous to that of animals (Burian et al. 2016; Lanfear 2018; Burian 2021). This proposed germline cell lineage is located in the shoot apical meristem (SAM) (Romberger et al. 1993; Kwiatkowska 2004; Watson et al. 2016). Remarkably, mitochondria in SAM meristematic cells exhibit dynamic fusion activity, leading to the formation of an extensive reticulate mitochondrial network (Segui-Simarro et al. 2008). The reticulate network facilitates the intermixing of mitochondrial genomes, thereby promoting recombination and gene conversion (Seguí-Simarro and Staehelin 2009; Rose and McCurdy 2017; Edwards et al. 2021). Such events can drive rapid shifts in heteroplasmy levels (Khakhlova and Bock 2006; Schaffner and Patel 2022), consequently promoting the segregation of heteroplasmic genomes. Notably, proteins involved in gene conversion are highly expressed in the SAM, supporting the active recombination and gene conversion processes (Edwards et al. 2021) (Box 3). Together, elevated mitochondrial fusion and genome recombination in the SAM may thus effectively mimic a bottleneck effect in plants.

Box 3. **MSH1, a promoter of vegetative segregation**

MSH1, initially identified as a “chloroplast mutator” in Arabidopsis thaliana, is later found to be critical for DNA repair, maintenance, and recombination surveillance in both plastids and mitochondria (Redei 1973; Sloan et al. 2024). Disruption of MSH1 results in a wide range of phenotypes affecting growth, developmental reprogramming, and stress responses (Xu et al. 2012; Virdi et al. 2016; Shao et al. 2017; Beltrán et al. 2018; Kundariya et al. 2020). Notably, recent findings highlight the role of MSH1 in gene conversion and recombination (Wu et al. 2020; Peñafiel-Ayala et al. 2024), processes that influence the sorting of heteroplasmic cytoplasmic genomes. This section focuses solely on MSH1's role in vegetative segregation. For a more comprehensive overview of its other functions, please refer to related reviews (Mackenzie and Kundariya 2020; Sloan et al. 2024).

In Arabidopsis, MSH1 facilitates efficient sorting of heteroplasmic genomes during plant development. Its role in mitochondrial genome segregation has been clearly demonstrated, as mitochondrial heteroplasmy is fixed more rapidly in wild-type plants compared to msh1 mutants. By mediating gene conversion, MSH1 promotes the homogenization of cytoplasmic genomes, thereby accelerating heteroplasmic sorting (Broz et al. 2022). Intriguingly, the loss of MSH1 function is also associated with increased substoichiometric shifting of mitochondrial genomes, leading to genome instability (Abdelnoor et al. 2003; Zhao et al. 2016). In addition, msh1 mutants exhibit increased inter-mitochondrial connectivity (Chustecki et al. 2022). This enhanced mitochondrial interaction promotes more frequent mixing of mitochondrial genomes, providing opportunities for illegitimate recombination and potentially exacerbating genome instability and heteroplasmy.

Given the multifaceted impacts caused by the loss of MSH1, it remains unclear whether the slower heteroplasmic sorting in msh1 mutants results directly from reduced MSH1-mediated gene conversion or indirectly from heightened genome instability. Nevertheless, these findings highlight the link between cytoplasmic genome maintenance and heteroplasmic sorting (Broz et al. 2022; Broz et al. 2024). The proposed role of gene conversion in generating variance presents a compelling model, offering an alternative mechanism for purifying selection in organisms without an early-segregated germline (Edwards et al. 2021; Veeraragavan et al. 2024).

While the gene conversion-dependent mechanism addresses mitochondrial heteroplasmy, it cannot resolve plastid heteroplasmy resulting from biparental inheritance. In such cases, distinct parental plastid genomes reside in separate plastids. Since plastids do not fuse, their genomes cannot intermix (Sears 1980; Seguí-Simarro and Staehelin 2009). Therefore, gene conversion cannot aid in the segregation of heteroplasmic plastid genomes. Instead, a bottleneck achieved by reducing plastid genome copy numbers may enhance segregation efficiency. In the SAM, plastid abundance and genome copy numbers are low, with amplification occurring during leaf development (Fujie et al. 1994; Greiner et al. 2020). This genome copy number dynamic mirrors the mitochondrial bottleneck observed during female germline development in animals (Shoubridge and Wai 2007; Stewart and Chinnery 2015).

Collectively, these findings suggest that plants employ diverse mechanisms to enforce bottleneck effects despite lacking a defined early-segregated germline (Edwards et al. 2021). A pronounced segregation of cytoplasmic genomes is observed between the stages of inflorescence formation and the transition to the next generation, indicating that reproductive tissues impose a tight bottleneck (Broz et al. 2024). Nevertheless, given the distinct dynamics of plastid and mitochondrial genomes, it is likely that the mechanisms driving their segregation differ substantially.

### The finish line—arriving at progeny gametes

Segregation of cytoplasmic genomes is a continuous and gradual process during plant development. Depending on the segregation dynamics, the heteroplasmy levels vary across cells, tissues, and organs, leading to distinct phenotypes even within the same plant.

A prime example is the coexistence of both female and hermaphrodite flowers within the same plant, caused by the differential sorting of mitochondrial genome variants (Erickson and Kemble 1993; Andersson 1999). Mutated mitochondrial genomes can cause cytoplasmic male sterility (CMS) by disrupting the formation of functional male gametes (Hanson 1991). Interestingly, CMS-inducing genomes are not strongly selected against in most tissues since they do not adversely affect somatic cell fitness (Budar and Pelletier 2001). Therefore, CMS-inducing and functional mitochondrial genomes undergo stochastic segregation and reach reproductive organs independently. This leads to the formation of “female flowers” with CMS-inducing genomes, while male-fertile hermaphrodite flowers develop in sectors with functional genomes (Erickson and Kemble 1993; Andersson 1999). Notably, these findings suggest that cytoplasmic genome segregation can occur rapidly. Heteroplasmic sorting is completed within a single generation with the formation of homoplasmic gametes (Broz et al. 2024). The cytoplasmic genome present in the homoplasmic gamete becomes the sole contributor to subsequent generations, emerging as the winner in this obstacle race. However, in species with less efficient segregation, gametes may still carry both paternal and maternal genomes, resulting in the persistence of heteroplasmy in the progeny (Bentley et al. 2010; Mandel et al. 2020). In such cases, the race between parental genomes continues into the next generation.

## Concluding remarks and future perspectives

By following this epic obstacle course race, we have witnessed how cytoplasmic genomes are transmitted across cells and generations. While numerous cellular processes have been proposed to regulate cytoplasmic inheritance, the specific genes and precise mechanisms underlying this process remain largely unidentified (see Outstanding Questions). This knowledge gap primarily stems from methodological limitations that have hindered our deeper understanding of cytoplasmic inheritance.

Historically, studies on cytoplasmic inheritance have relied on molecular techniques such as restriction fragment length polymorphism analysis. While these methods, combined with cytological studies, provided foundational insights, they also present significant limitations that may lead to a biased view of cytoplasmic inheritance. Notably, studies using these techniques were conducted at restricted sample sizes, making it difficult to detect rare leakage events. Furthermore, their sensitivity was often insufficient to identify small amounts of DNA, leading to an underestimation of paternal leakage and heteroplasmy (Reboud and Zeyl 1994; Hagemann 2004). Another major limitation is the lack of discernable phenotypic markers that distinguish between paternal and maternal genomes (Reboud and Zeyl 1994), which restricts the feasibility of developing large-scale screenings for studying cytoplasmic inheritance.

Emerging technological advancements offer solutions to these challenges. For instance, plastid genome engineering enables the expression of selectable markers and fluorescent reporters within plastids, facilitating reliable and highly sensitive screening for paternal plastid inheritance (Ruf et al. 2007; Chung et al. 2023). Recent breakthroughs in mitochondrial genome engineering further revolutionize this field by enabling the design of distinctive mitochondrial genomes (Maliga 2022; Arimura and Nakazato 2024). However, stable mitochondrial genome transformation through transgene insertion is not yet achievable in plants, limiting the development of selectable markers and reporter systems similar to those used in plastid inheritance research. Nevertheless, precise mitochondrial DNA base-editing, coupled with advanced sequencing and PCR techniques, allows for the quantification of heteroplasmic genomes at the single-cell level (Kotrys et al. 2024). Additionally, single-molecule genome tracking technique presents unprecedented opportunities to study vegetative segregation and partitioning of cytoplasmic genomes during cell division (Li et al. 2023; Roussou et al. 2024).

By leveraging these advanced research tools, we will be able to identify key regulatory genes and mechanisms underlying cytoplasmic inheritance. A comprehensive understanding of these cellular processes will pave the way for targeted manipulation of cytoplasmic inheritance. Since many agronomically important traits are influenced by cytoplasmic genomes, controlling cytoplasmic inheritance holds transformative potential for plant breeding.

**Advances box**

- New genes have been identified as key regulators of cytoplasmic inheritance in plants and algae, providing compelling evidence supporting the role of organelle quality control and cytoplasmic genome maintenance in governing cytoplasmic inheritance.
- By manipulating growth conditions and genetic background, the uniparental inheritance of cytoplasmic genomes can be disrupted, consequently leading to the generation of heteroplasmic progeny.
- Advances in cytoplasmic genome engineering and single-molecule imaging offer powerful tools for studying the vertical transmission and vegetative segregation of cytoplasmic genomes, enabling the design of large-scale screenings and real-time in vivo tracking of heteroplasmy.
- With more sensitive methods for detecting cytoplasmic genome variants, the perceived rarity of heteroplasmy due to technical detection limitations will be overcome. As evidence of paternal leakage and biparental inheritance continues to accumulate, the long-standing notion of maternal inheritance as the norm will be challenged.

**Outstanding questions**

- Interspecific crosses involve the mating of individuals from different species, which often leads to a disruption in the uniparental inheritance of cytoplasmic genomes. What are the mechanisms responsible for the breakdown of uniparental inheritance in interspecific crosses?
- The mode of cytoplasmic inheritance (maternal, paternal, or biparental) has exhibited remarkable lability among plant species. For instance, maternal inheritance is predominant in angiosperms, whereas paternal inheritance is commonly observed in gymnosperms. What drives this evolutionary flexibility in inheritance patterns across plant lineages? What genetic, molecular, and physiological factors contribute to this phenomenon?
- To date, only a limited number of genes have been identified as regulators of cytoplasmic inheritance. What other genes and factors play a role in regulating this process?
- To what extent can we manipulate cytoplasmic inheritance patterns? How can we manipulate cytoplasmic inheritance to improve crop production?

## Data Availability

There are no new data associated with this article.

## Dive Curated Terms

The following phenotypic, genotypic, and functional terms are of significance to the work described in this paper:

MSH1 Gramene: AT3G24320
MSH1 Araport: AT3G24320

OSB1 Gramene: AT1G47720
OSB1 Araport: AT1G47720

RECA3 Gramene: AT3G10140
RECA3 Araport: AT3G10140

dpd1 Gramene: AT5G26940
dpd1 Araport: AT5G26940

WHIRLY2 Gramene: AT1G71260
WHIRLY2 Araport: AT1G71260
